# Optimizing subjective wellbeing with amisulpride in first episode schizophrenia or related disorders

**DOI:** 10.1017/S0033291722003142

**Published:** 2023-10

**Authors:** Lieuwe de Haan, Mirjam van Tricht, Floor van Dijk, Celso Arango, Covadonga M. Díaz-Caneja, Julio Bobes, Leticia García-Álvarez, Stefan Leucht

**Affiliations:** 1 Department of Psychiatry, Amsterdam UMC, Location AMC, University of Amsterdam, Amsterdam, the Netherlands; 2 Arkin, Institute for Mental Health, Amsterdam, the Netherlands; 3 Department of Child and Adolescent Psychiatry, Institute of Psychiatry and Mental Health, Hospital General Universitario Gregorio Marañón, IiSGM, CIBERSAM, School of Medicine, Universidad Complutense, Madrid, Spain; 4 Department of Psychiatry, University of Oviedo, Instituto de Investigación Biosanitaria del Principado de Asturias (ISPA), INEUROPA, CIBERSAM. Oviedo, Spain; 5 Department of Psychiatry and Psychotherapy, Technical University Munich, Munich, Germany

**Keywords:** schizophrenia, antipsychotic, subjective wellbeing, response, amisulpiride

## Abstract

**Background:**

Subjective response (SR) to antipsychotic medication is relevant for quality of life, adherence and recovery. Here, we evaluate (1) the extent of variation in SR in patients using a single antipsychotic; (2) the association between subjective and symptomatic response; and (3) predictors of SR.

**Methods:**

Open-label, single treatment condition with amisulpride in 339 patients with a first episode of a schizophrenia spectrum disorder, at most minimally treated before inclusion. Patients were evaluated at baseline, before start with amisulpride and after four weeks of treatment with the Subjective Wellbeing under Neuroleptic scale, the Positive and Negative Syndrome Scale, and the Calgary Depression Scale for Schizophrenia.

**Results:**

(1) 26.8% of the patients had a substantial favorable SR, and 12.4% of the patients experienced a substantial dysphoric SR during treatment with amisulpride. (2) Modest positive associations were found between SR and 4 weeks change on symptom subscales (*r* = 0.268–0.390, *p* values < 0.001). (3) Baseline affective symptoms contributed to the prediction of subjective remission, demographic characteristics did not. Lower start dosage of amisulpride was associated with a more favorable SR (*r* = −0.215, *p* < 0.001).

**Conclusions:**

We conclude that variation in individual proneness for an unfavorable SR is substantial and only modestly associated with symptomatic response. We need earlier identification of those most at risk for unfavorable SR and research into interventions to improve SR to antipsychotic medication in those at risk.

Subjective wellbeing of patients with schizophrenia or related disorders is an independent outcome measure relevant for quality of life, adherence and recovery (de Haan, Nimwegen, Amelsvoort, Dingemans, & Linszen, 2008; Karow et al., 2007; Lambert et al., 2007; Naber et al., 2001). Subjective wellbeing is strongly associated with subjective quality of life and can be reliably reported by the vast majority of first episode patients (de Haan, Weisfelt, Dingemans, Linszen, & Wouters, 2002). Patients with schizophrenia or related disorders experience a substantially decreased subjective wellbeing compared to their siblings and healthy controls (Vothknecht et al., 2013). Improving subjective wellbeing in patients with schizophrenia is therefore a therapeutic goal in its own. Subjective wellbeing is partially overlapping with concepts as quality of life or personal recovery. However, subjective wellbeing is more restricted to self-reported evaluation of own mental functioning and affective state, while quality of live or personal recovery measures also include aspects of connectedness with others and social functioning. Antipsychotic medication influences subjective wellbeing (Naber, 1995; Vothknecht, Schoevers, & de Haan, 2011). For instance, antipsychotic medication leading to a dopamine D_2_ occupancy of higher than 70% is associated with subjective un-wellbeing in imaging studies (de Haan, Lavalaye, Linszen, Dingemans, & Booij, 2000, 2003; Mizrahi et al., 2007) and in daily life (Lataster et al., 2011). Especially, patient's early experience of the impact of antipsychotic medication on their wellbeing is relevant. It has been found that subjective response (SR) to 4 weeks of treatment with antipsychotic medication impacts on medication compliance and predicts remission after 5 years (de Haan et al., 2008; Karow et al., 2007). SR refers to the change in patient's evaluation of his/her mental state during the first 4 weeks of treatment.

As recommended by the Remission in Schizophrenia Working Group, the components of remission should include subjective quality of life or subjective remission, next to symptomatic and functional remission (Andreasen et al., 2005). Subjective remission refers to a state in which subjective wellbeing is good enough and further improvement is not a priority. Improving multimodal remission is an important clinical challenge. The importance of the concept multimodal remission is also illustrated by Castelein, Timmerman, PHAMOUS investigators, van der Gaag, and Visser (2021) who found that happiness of patients with schizophrenia spectrum disorder was partly independent of societal or symptomatic recovery. Of note, only 13% of treated schizophrenia outpatients met symptomatic, functional and subjective well-being remission criteria after approximately the first five years of treatment (Lambert et al., 2006).

Further, the clinical relevance of the concept SR or subjective remission depends on three unanswered questions [1] the extent of variation in SR or subjective remission independent of type of antipsychotic medication, [2] its relative independence from symptomatic response and remission, and on [3] identifiable predictors of SR or subjective remission. We will describe the background of these questions below.

The first question we will try to answer in the current paper is: is there clinically relevant variation in SR or subjective remission between patients treated with the same antipsychotic drug? Although substantial variation in SR has been found in earlier studies, it has not yet been investigated whether this variation also exists in first episode patients all treated with the same antipsychotic drug. Thus, it is still unknown what the impact is of individual patient characteristics on the variation in subjective wellbeing, and which part of the variation is caused by other factors, such as type or dose of antipsychotic drug. Especially when an antipsychotic drug has a favorable balance between effect and adverse effect, variation in SR or subjective remission under these conditions refers to the potential importance of patient factors. Amisulpride is such an agent, it is a selective D_2_/D_3_ receptor antagonist with mesolimbic selectivity (Schoemaker et al., 1997), high efficacy and low risk for metabolic (except prolactin) and extrapyramidal side-effects relative to other antipsychotic agents (Davis, Chen, & Glick, 2003; Geddes, Freemantle, Harrison, & Bebbington, 2000; Huhn et al., 2019; Kahn et al., 2018). Therefore, the OPTiMiSE study – a study that focuses on optimizing treatment within a relatively homogeneous sample of first episode patients all treated with amisulpride – offers an unique possibility to elucidate the extent of variation in SR or subjective remission not attributable to differences in type of antipsychotic medication.

The second question is: To what extent is variation in SR or subjective remission associated with symptomatic response or remission? When subjective and symptomatic outcome co-vary, focus on symptomatic response or remission would be sufficient to capture variation between patients. However, there are indications that SR and subjective remission are semi-independent of symptom changes (Naber, 1995). It is still unknown to what extent co-variation between SR or subjective remission and symptomatic response or remission occurs in a relatively homogeneous group of patients treated with the same antipsychotic medication. Evidence for the relative independence of SR or subjective remission from symptomatic response would support the clinical relevance of subjective outcome measures in the pharmacological treatment of schizophrenia spectrum disorders.

The third question we will try to answer is: which demographical or clinical characteristics at baseline predict SR or subjective remission? Although some studies found evidence for an association of type and dose of medication with SR, not all studies report this association (de Wit, van Dijk, Meijer, van Tricht, & de Haan, 2017). We therefore investigated whether starting dose of amisulpiride predicts SR. Moreover, SR on antipsychotic medication is not only related to factors associated with medication but there are indications that a substantial part of the variation in SR is related to demographical or clinical differences between patients (Pos et al., 2017; van Dijk, Schirmbeck, & Haan, 2018). Knowledge concerning individual risk factors for an unfavorable or dysphoric response would enable patient selection for specific strategies to ameliorate SR.

## Methods

For a detailed description of methods of the Optimise study we refer to Leucht et al. (2015), Kahn et al. (2018) and ClinicalTrials.gov, number NCT01248195. Below we will give a global overview.

### Design

The multinational and multicenter Optimise study comprised a combination of treatment designs. Here we only report on the first phase of the study concerning an open-label, single treatment condition with amisulpride.

### Patients

Patients with a first episode of schizophrenia, schizophreniform, or schizoaffective disorder according to DSM-IV, aged between 18 and 40 years, who have given written informed consent were included. Participants had a maximum interval between the onset of psychosis and study entry of 2 years and had used antipsychotic medication for no longer than 2 weeks in the previous year or 6 weeks lifetime, before inclusion.

### Procedures

After patients signed informed consent, the screening visit was done, during which eligibility was assessed and baseline data collected. For this paper, we only used data of the baseline and 4 weeks assessment of the Positive and Negative Syndrome Scale; PANSS), Calgary Depression Scale for Schizophrenia; CDSS), and the Subjective Wellbeing under Neuroleptic scale; SWN), The SWN (Naber, 1995) is a self-rating scale with good validity and reliability (de Haan et al., 2002). The SWN is sensitive to the influence of antipsychotic medication (Vothknecht et al., 2011, 2013) and it is used to assess self-reported quality of life concerning the past 7 days. The SWN consists of 20 statements (10 positive and 10 negative) with a minimum total score of 20 (indicating low subjective wellbeing) and a maximum total score of 120 (indicating good subjective wellbeing). Each item is rated on a Likert scale (1–6).

SR is defined as change in total SWN score from baseline to 4 weeks. Clinically relevant change in subjective wellbeing is defined as a change of 10 or more in the SWN total score (Lambert et al., 2006, 2007). An increase of 10 or more may be considered a clinically relevant favorable SR, while a decrease of 10 or more may be considered a clinically relevant unfavorable (or dysphoric) SR. The concept subjective remission is defined as a score of 80 or more on the SWN (Lambert et al., 2007). A total score of 80 corresponds to an average rating of markedly positive subjective wellbeing concerning the positive SWN items and ratings of only mildly impairment concerning the negative SWN items, and indicates an adequate subjective wellbeing.

Symptomatic remission is defined according to the criteria of Andreasen et al. (2005): eight symptoms of schizophrenia as measured by the PANSS (Kay, Fiszbein, & Opler, 1987) (PANSS items P1, P2, P3, N1, N4, N6, G5, and G9) at the most only mildly present, implying that symptoms do not interfere with daily life functioning.

Symptomatic response is defined as PANSS total score change from baseline. In addition, symptomatic response was measured using score changes on subscales on the PANSS, according to the factor model of van der Gaag et al. (2006; i.e. positive symptoms, negative symptoms, disorganization, excitement and emotional distress).

### Statistical analysis

Variation in SR was expressed by calculating mean, standard deviation (s.d.) and ranges. Pearson correlation analyses were used to determine associations of SR with symptomatic response (i.e. difference scores between baseline and four weeks on PANSS and CDSS scales). We used linear regression analyses to determine if variation in clinical scale scores and demographical (i.e. age, gender, years of education) characteristics at baseline predict variation in SWN total score at four weeks. Associations of amisulpride start dosages with SR were tested using Pearsons' correlation coefficients. Moreover, logistic regression analyses were conducted to investigate whether baseline clinical and demographic variables predicted clinically relevant improvement in subjective wellbeing (i.e. total change in the SWN of 10 or more = improvement; change of 10 or less is no improvement) or subjective remission (i.e. score in the SWN of 80 or more = remission, score of > 80 = no remission) status at the four-week follow-up.

## Results

446 patients met inclusion criteria and initiated the first open label treatment with amisulpride (200 − 800 mg/day). Mean age was 25.8 (s.d. 6.0), 30.3% were female. 121 individuals were not in symptomatic remission at the end of phase 1. An assessment of subjective experience before the start with amisulpiride and after 4 weeks treatment with amisulpiride was available for 339 patients.

### Is there clinical relevant variation in SR or subjective remission between patients treated with amisulpride?

**Fig. 1.**
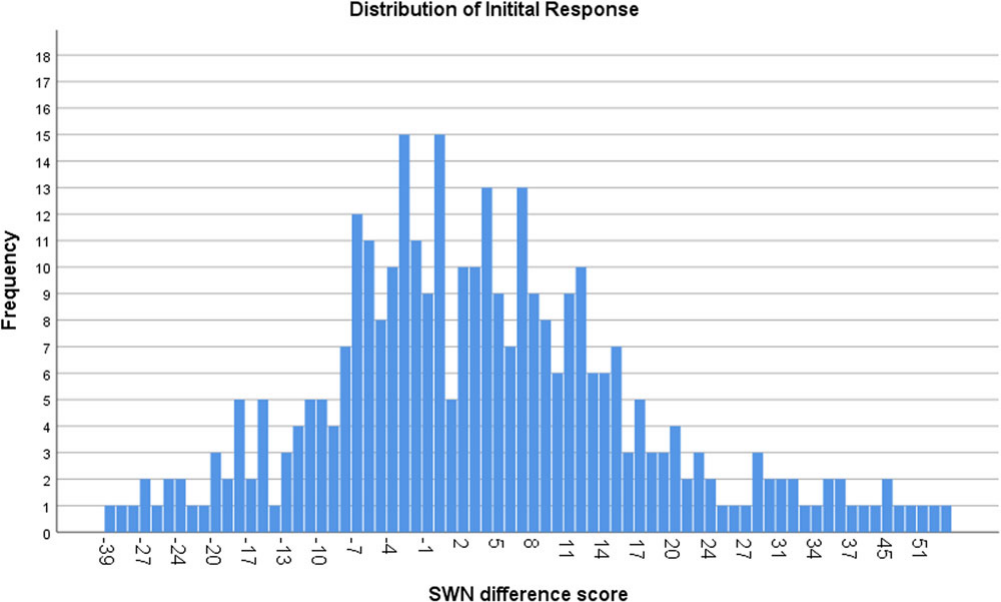
Distribution of initial subjective response.

Clinically relevant SR, defined as an increase of 10 points or more on the SWN-total score, was observed in 91 out of 339 subjects [26.8% of the patients]. However, 42 out of 339 subjects [12.4% of the patients] experienced a clinically relevant initial unfavorable or dysphoric response during treatment with amisulpride, defined as a decrease in total SWN score of 10 or more. Variation in initial SR scores is presented in Fig. 1.

Subjective remission, defined as a SWN total score of 80 or higher, was present in 244 out of 339 subjects [72.0% of the patients] after 4 weeks of treatment with amisulpride.

### To what extent is variation in SR or subjective remission associated with symptomatic improvement?

We found modest but significant associations between SWN difference score and symptomatic change on all PANSS subscales (*r* = 0.268–0.390, all *p* values < 0.001). Moreover, changes in SWN scores from baseline to 4 weeks treatment were associated with changes on the CDSS (*r* = 0.330, *p* < 0.001). Meaning, that improvement of subjective experience is modestly associated with improvement of PANSS subscales and CDSS.

Linear regression analyses yielded that only a small percentage of variation in 4-week change in the SWN total score (i.e. SR) is explained by 4-week score changes on the PANSS subscales (*R*^2^ = 0.18). Subsequent analyses showed that 4-week score changes on the PANSS positive (*B* = 0.385, *t* = 1.994, *p* = 0.047), negative (*B* = 0.329, *t* = 2.025, *p* = 0.044), emotional distress (*B* = 0.78, *t* = 4.162, *p* < 0.001) and excitement (*B* = −0.575, *t* = −2.615, *p* = 0.009) subscales significantly, but modestly, contributed to the prediction of variation in SR.

The overlap between subjective and symptomatic remission four weeks after treatment is presented in Table 1.

**Table 1.** The overlap between subjective and symptomatic remission four weeks after treatment

	Subjective remission after 4 weeks		Total
	Yes	No	
Symptomatic remission after 4 weeks			
Yes	193	39	232
No	54	53	107
Total	247	92	339

### Which demographic and clinical characteristics at baseline predict SR or subjective remission?

Demographic characteristics (age, gender, years of education) did not contribute to the prediction of SR or subjective remission. Moreover, independent sample *t* test yielded no significant difference in SWN scores at baseline or after 4 weeks treatment, nor were there significant differences found in SWN difference scores between men and women in our sample (all *p* values > 0.579).

Logistic regression analyses yielded that baseline scores on the PANSS subscale emotional distress (Wald = 4.10, *p* = 0.043, Exp (*B*) = 1.073) and the CDSS total score (Wald = 16.12, *p* < 0.001, Exp (*B*) = 1.167) contributed to the prediction of subjective remission status after 4 weeks of treatment with amisulpiride. However, PANSS and CDSS scores at baseline did not significantly contribute to the prediction of SR (i.e. improvement of > 10 on the SWN *v.* no improvement). We found that a lower start dosage of amisulpride (mean: 297.6, s.d.: 246.1) was associated with a higher SR (*r* = −0.215, *p* < 0.001).

Since we found an association between start dosage of amisulpiride and subjective improvement in 4 weeks treatment we performed a posthoc analysis whether we could preliminary identify if a certain start-dosage drives this finding. We found that a start dosage of 600 mg or more was associated with less subjective improvement than start dosages of 600 mg or less.

Moreover, we also found a small, negative association of amisulpride dosages at 4 weeks treatment (mean: 441.5, s.d.: 212.9) with SR (*r* = −114, *p* = 0.040).

## Discussion

### Clinical relevant variation in SR or subjective remission between patients treated with amisulpride

Our findings indicate that there is substantial and clinical relevant variation in SR and subjective remission among first episode patients with schizophrenia spectrum disorders treated with amisulpride. More than a quarter of the patients experience a substantial improvement of subjective wellbeing in the first 4 weeks of treatment with amisulpiride. On the other hand, about one out of eight patients experience a substantial dysphoric response after 4 weeks treatment with amisulpride. This variation in SR is also reflected in the 28% of patients not achieving subjective remission after 4 weeks treatment. Since subjective quality of life is an important treatment goal these results are disturbing. A substantial part of first episode patients deteriorates during treatment or does not reach subjective remission. Clinical relevance of variation in SR is also illustrated by the earlier found associations of SR with medication compliance, recovery, symptomatic and functional remission (de Haan, van Amelsvoort, Dingemans, & Linszen, 2007, 2008; Lambert et al., 2006).

First, we will compare our finding of substantial variation in SR in first-episode patients all treated with amisulpride, with results from previous studies. Most relevant in this case is the study by Lambert et al. (2006), who found in a large multicenter observational study that 70% of the patients all treated with amisulpride had a favorable SR in the first 4 weeks. The other 30% of the patients were classified as a SWN non-responder (i.e. their mean SWN score increased only little and non-significantly from 49.6 to 53.3). So, the subgroup that does not reach clinical relevant favorable SR is comparable in both studies. However, a more substantial proportion of the patients in the Lambert study experience clinical relevant improvement of subjective wellbeing. An important difference between our study and the study of Lambert et al., is that in their study most patients received several other antipsychotic agents before treatment with amisulpiride. Possibly, amisulpiride improves wellbeing more substantially after a switch from other antipsychotic agents. In a double blind randomized controlled study comparing the SR of olanzapine and clozapine, a mean improvement of subjective wellbeing was found of 11.3 points with a considerable standard deviation of 20.7 in the olanzapine condition and a mean improvement of 8.2 with also a substantial standard deviation of 15.8 in the clozapine condition (Naber et al., 2005). These findings are comparable to ours by also showing that variation in SR is substantial in patients treated with other antipsychotic agents as we find in patients treated with amisulpiride.

Second, we will compare our finding of subjective non-remission in 28% of the patients with results from previous studies. We already mentioned that Lambert found a comparable figure of 30% of amisulpiride treated patients not reaching subjective remission. From the double blind randomized comparison between olanzapine and risperidone (Naber et al., 2005) we estimated that approximately 50% of the patients do not reach subjective remission (figure 2 on page 111). This more unfavorable percentage of subjective non remission may be explained by characteristics of the participants in the study of Naber et al. (2005) and Lambert et al. (2009): mean number of previous psychotic episodes was 4.5 and patients either failed to respond to at least one antipsychotic agent (other than olanzapine or clozapine) or experienced intolerable side effects during prior antipsychotic treatment. In contrast, patients included in our study experienced their first psychotic episode and were not or only minimally treated with antipsychotic medication before inclusion.

### Modest association between variation in SR or subjective remission and symptomatic improvement

In answer to our second question, we found that although variation in SR and subjective remission is to some extent associated with symptomatic improvement, the most substantial part of variation in SR and subjective remission is not explained by variation in symptomatic response. From the distinct symptom domains, emotional distress showed the strongest association with SR. Taken together, this means that it is not enough to measure symptomatic response alone to evaluate the impact of antipsychotic medication on subjective wellbeing. Assessment of SR is also necessary. These findings are concordant with conclusions from several earlier reports (Lambert et al., 2007; Naber et al., 2001, 2005).

### Baseline demographical or clinical characteristics do not substantially predict SR or subjective remission

The demographic characteristics we evaluated did not contribute to the prediction of SR or subjective remission. From the baseline clinical characteristics, only severity of emotional distress and depressive symptoms were significantly associated with SR. Apparently, those with more emotional distress or depressive symptoms have a higher propensity to experience a dysphoric SR during the first 4 weeks of treatment with amisulpiride. These findings are partly in line with earlier findings (Lambert et al., 2007). As in our study, no associations between demographic characteristics and SR were found in an earlier study of patients treated with amisulpride (Lambert et al., 2007). Nevertheless, they found small size associations between positive and negative symptoms and SR. Higher baseline positive symptoms score predicted slightly higher odds (1.06) of favorable SR and higher baseline negative symptoms score predicted slightly lower odds (0.96) of favorable SR. Severity of emotional distress was not assessed in their study and severity of depressive symptoms was not significantly associated with SR. Probably, our sample was too small to find this very subtle (and clinically non-relevant) association between symptom severity of positive or negative symptoms and SR. Mostly in line with our results, in a very large observational study (2345 participants) both baseline demographic characteristics (age, gender) and clinical characteristics (global severity of positive, negative, cognitive or depressive symptoms) did not significantly predict subjective wellbeing (Lambert et al., 2006). It is worth mentioning that in this study independent living was a substantial predictor of reaching adequate subjective wellbeing (OR = 1.53) as was first antipsychotic treatment (OR = 1.49) and typical antipsychotic agents (OR = 0.57). These factors also appeared to be relevant predictors of stable clusters of subjective wellbeing during three years in another report concerning the same sample (Lambert et al., 2009). The finding in their study that first episode patients tend to have more substantial improvement in subjective wellbeing than multi-episode patients underscores the importance of our finding that as much as one out of eight first episode patients experience substantial dysphoria during treatment with a first choice antipsychotic drug.

In line with earlier findings (de Haan et al., 2000, 2003; Lataster et al., 2011; Mizrahi et al., 2007) we found an association between start dose of treatment with amisulpiride and SR, implying that higher initial dopamine D_2_ occupancy by antipsychotic medication is associated with higher odds on a dysphoric response. Especially initial dosage higher than 600 mg was associated with less subjective improvement. Although current study was not designed to evaluate the optimal start dosage and although it is questionable whether the post hoc statistical analysis is warranted, we think that this finding preliminary indicates that from a ‘effect on subjective experience’ perspective, the initial dosage of amisulpiride should be lower than 600 mg.

Since SR is clinical very relevant and for a substantial part independent from symptomatic response it is important to evaluate which factors predict SR. We already know that dose and type of antipsychotic medication has an impact on SR. In our study we showed that variation in individual vulnerability for unfavorable SR or subjective remission is substantial. Taken together, neither demographic nor baseline clinical variables are robust predictors of SR or subjective remission. Therefore, the question remains which patient or patient-drug factors predict SR or subjective remission. Therefore, we need to find out whether other patient factors or patient-drug interactions are associated with SR and subjective remission. Given the clinical relevance of SR we need this information urgently. Possible candidates are genetic variation, profile of proteomics, characteristics of dopaminergic neurotransmission, pharmacokinetics or psychological characteristics.

### Strengths and limitations

We consider the following characteristics of the current study as strengths. All patients suffered from a first episode of psychosis with a schizophrenia spectrum diagnosis and had a history of none or minimal antipsychotic medication use. Therefore, their response to antipsychotic medication was not confounded by the effects of substantial earlier antipsychotic treatment. Moreover, the same antipsychotic with a favorable effect/adverse effect balance was used for all patients, offering an ideal opportunity to disentangle variation in SR associated with differences between patients.

We also need to acknowledge several limitations. Most importantly assessment of SR at baseline and after 4 weeks was missing in 26% of the included patients. Moreover, a more detailed assessment of individual characteristics at baseline is necessary to evaluate whether we can predict who will develop an unfavorable SR and who will not. Finally, only patients capable and willing to sign informed consent, and treated voluntarily were included. This means that the sample we studied is not representative for all patients with first episode psychosis.

### Clinical and research implications

We conclude that individual variation in vulnerability for showing non-favorable SR and not achieving subjective remission during the first weeks of treatment after a first episode of psychosis is substantial and largely independent from symptomatic response. Lower starting dose of amisulpride was associated with more favorable SR. Neither demographic, nor baseline clinical variables are robust predictors of SR or subjective remission in our sample (although we did find a relationship between severity of baseline affective symptoms and not achieving subjective remission). Since SR and subjective remission are relevant for quality of life, adherence and recovery, further research including additional variables is needed to identify factors that predict SR and subjective remission in the early stages after a first episode of psychosis, to enable an earlier identification of those most at risk for unfavorable SR or non-SR. Moreover, we need research into interventions to improve SR and subjective remission in those at risk.
